# Flexibility of Older Adults Aged 55–86 Years and the Influence of Physical Activity

**DOI:** 10.1155/2013/743843

**Published:** 2013-06-19

**Authors:** Liza Stathokostas, Matthew W. McDonald, Robert M. D. Little, Donald H. Paterson

**Affiliations:** ^1^Canadian Centre for Activity and Aging, Faculty of Health Sciences, 3M Centre 2225, The University of Western Ontario, London, ON, Canada N6A 3K7; ^2^School of Kinesiology, Faculty of Health Sciences, 3M Centre 2225, The University of Western Ontario, London, ON, Canada N6A 3K7

## Abstract

Cross-sectional age-related differences in flexibility of older adults aged 55–86 years of varying activity levels were examined. Shoulder abduction and hip flexion flexibility measurements were obtained from 436 individuals (205 men, 71 ± 9 years; 231 women, 72 ± 8 years). Total physical activity was assessed using the Minnesota Leisure-Time Physical Activity Questionnaire. Shoulder abduction showed a significant decline averaging 5 degrees/decade in men and 6 degrees/decade in women. Piecewise linear regression showed an accelerated decline in men starting at the age of 71 years of 0.80 degrees/year, whereas in women the onset of decline (0.74 degrees/year) was 63 years. Men and women showed a significant decline in hip flexion (men: 6 degrees/decade; women: 7 degrees/decade). Piecewise linear regression revealed a rate of decline of 1.16 degrees/year beginning at 71 years in men and in women a single linear decline of 0.66 degrees/year. Multiple regression analysis showed that age and physical activity accounted for only 9% of the variance in hip flexion in women and 10% in men, with age but not physical activity remaining significant. Similarly for shoulder abduction, age was significant but not physical activity, in a model that described 8% of the variance for both sexes.

## 1. Introduction

As indicated in a recent systematic review by our group [1], there is conflicting information regarding both the relationship between flexibility training interventions and functional outcomes and the relationship between improved flexibility and daily functioning; health benefits have not yet been established. The comparison of studies in this area to provide a prescription of the flexibility is complicated by the variety of limb ranges of motion studied, testing procedures utilized, and methods of assessing physical activity levels. Furthermore, this component of physical health has been somewhat neglected or forgotten in the current literature despite the lack of evidence for recommendations of the amount and type of flexibility needed for health in older adults. Further, despite this lack of a synthesis of the literature to support the recommendation of the inclusion of a flexibility component to older adult exercise programs, many older adult activity programs place a considerable emphasis on flexibility. The present study attempts to add additional insight to this area by presenting the relationship between declines in flexibility across age and functional outcomes in a large sample of individuals representing the older adult age range. Joint flexibility may decrease across the age span [2–4], which has the potential to affect normal daily functioning. Upper body flexibility is known to be important for activities such as getting dressed and reaching for objects, while lower body flexibility is important for maintaining normal walking patterns and for activities involving bending and reaching [5]. While the loss in flexibility with age has been attributable, in part, to decreased activity [5], the literature describing the influence of physical activity on flexibility and the aging process is surprisingly limited. The purpose of the present study was to examine the cross-sectional age-related differences in flexibility in a large sample of independently living adults aged 55–86 years with varying activity levels. The recent systematic literature review identified the lack of an established relationship between improved flexibility and daily functioning and health benefits [1]. As such, a secondary purpose of the present study was to describe any relationships of physical activity levels and of functional outcomes (specifically walking), with flexibility measures.

## 2. Methods

### 2.1. Sample Selection, Recruitment, and Participation

 The municipal tax assessment list, containing names of householders and residents in the city of London, Ontario (2011, population 366,151), provided the sampling frame. Those living in institutions, defined as nursing homes or chronic care facilities, were not eligible. A stratified random sample was drawn from the population. The strata were defined by gender and six five-year age groups, starting with age of 55, and the sampling rate was set to select 35 men and women in each stratum. Those sampled were sent a letter inviting their participation, and a follow-up call to recruit and screen the respondents was made. Exclusion criteria were those who responded no to the question of their ability to walk an 80 meter course. Thus, the target population the noninstitutionalized population aged 55–85 years who self-reported the ability to walk 80 meters. The university's human research review board approved the study, and each subject signed informed consent.

### 2.2. Measurements

#### 2.2.1. Anthropometric

 Body height, mass, skinfold thickness (four sites: biceps, triceps, subscapular, and suprailiac), and waist and hip girth were measured. Body mass index was calculated.

#### 2.2.2. Joint Flexibility

 Hip flexion was assessed using a Leighton flexometer fastened to the hip, with the range of motion determined by bending backward as far as possible and then forward as far as possible [6]. Shoulder abduction was measured as the range of motion of the right hand from the side of the leg, upward and outward in an arc [6].

#### 2.2.3. Physical Activity

 The Minnesota Leisure-Time Physical Activity Questionnaire (MLTPAQ) was used to assess self-reported physical activity levels [7]. The questionnaire was administered with the aid of a research assistant. For the present data in older adults, the intensity codes (metabolic rate scores) for different activities, developed for middle-aged subjects, were reduced in proportion to the age-related slowing of self-paced walking speed, to acknowledge that older adults would pursue these activities at a slower absolute pace. Specifically, the codes were decreased 8.9% for males and 4.6% for females from middle age to 55 years [8]. The codes were then decreased a further 0.61% per year in males and 1.02% per year in females for each year beyond the age of 55, corresponding to the rate of decline in self-paced walking as measured in the sample of the present study (age 55 to 85 years). Thus, the “intensity codes” for the vigor with which older adults participated in a particular activity were age-adjusted. According to the MLTPAQ scoring system, physical activity was characterized by total energy expenditure (in MET/minutes/day) and also examined for energy expenditure in activities characterized as light, moderate, or heavy intensity.

#### 2.2.4. Muscle Strength

 The design of the leg dynamometer and the procedures followed for measuring plantar flexion strength have been previously reported [9]. Subjects were seated on a bench with the thigh locked in a horizontal position and knee flexed at 85 degrees in the leg dynamometer. The dominant leg was clamped down and the subject was asked to push-off, that is, to attempt to raise their heel off the ground. The force generated against the clamp bar was recorded with a strain gauge calibrated with standard weights. Three trials were done. Maximal grip strength (of three trials) of the dominant hand was measured using a handgrip dynamometer with interchangeable casings to accommodate hand size.

#### 2.2.5. Normal and Fast Walking

 As a measure of lower body function, walking speed (time/meters) and step length were assessed by having subjects walk an 80-meter course at their normal and fast self-selected speeds [8].

#### 2.2.6. Self-Rated Health and Life Satisfaction

 Self-rated health and life satisfaction were assessed using a questionnaire containing modified questions from the Nottingham Health Profile [10], as were self-reported walking difficulty and difficulty with stairs, by rating degree of difficulty on a five-point scale.

### 2.3. Analysis

Data analyses were performed with the Statistical Package for the Social Sciences (SPSS 19.0, Ireland, 2010). All descriptive data are presented as mean ± SD. Frequency distributions were examined for categorical variables. Ranges of motion of the hip and shoulder joints across age were analyzed by both linear regression and piecewise linear regression with a 2-segment model (Sigma Plot 12.0, Chicago, Illinois, USA); these fits produced similar *R* ^2^ values. Age and physical activity were entered into a multiple regression analysis to determine associations with shoulder and hip flexibility. Further, expanded univariate logistic regression was performed to identify other variables associated with determining flexibility. Lastly, stepwise linear regression, allowing for entry and removal at the 0.10 level of significance, was used to examine the relationship of flexibility with physical (self-rated health, self-reported arthritis, body mass index, and upper and lower body strength), functional (walking and stair climbing difficulty, step length, and walking speed), and psychosocial (self-rated health, life satisfaction) variables.

## 3. Results

### 3.1. Response Rate

The recruitment process resulted in 1451 individuals contacted; 696 were eligible, and 441 (63.4%) participated and is detailed by Koval et al. [11]. Participants were more likely to be widowed and less likely to be married, more likely to have had a white-collar job, and had some postsecondary education.

### 3.2. Description of the Sample

Flexibility measurements were obtained from a total of 436 community-dwelling individuals (205 men, mean age 70.4 ± 8.8 years; 231 women, mean age 71.4 ± 8.4 years). Subject characteristics are presented in Table 1. Self-rated health among the group indicated that 11% of the sample considered their health to be “excellent” and 54% rated their health as “good.” Almost half of the sample was fully retired (49%) and 11% were employed full time. Seven percent of the sample reported being not “very satisfied” with life. Sixty-three reported being “quite satisfied” and 30% reported being “very satisfied.” The marital status of the sample indicated that 56% were married. Based on self-reported physical activity levels, the calculated total energy expenditure in leisure time physical activity would indicate that the present sample was, on average, very active, but encompassed a wide range of activity levels.

### 3.3. Flexibility and Differences by Age

#### 3.3.1. Shoulder Abduction

**Figure 1:**
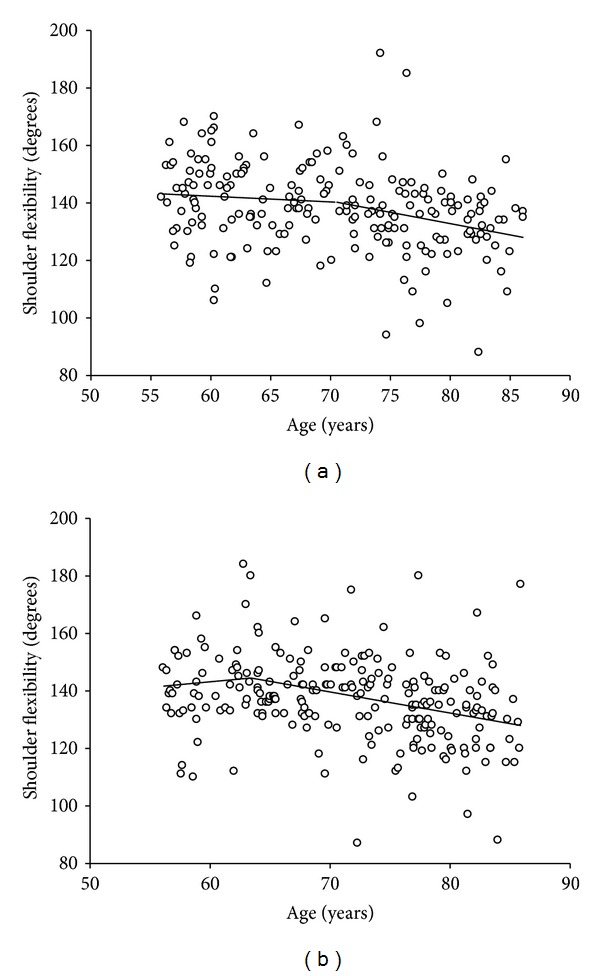
(a) Age analysis for shoulder flexibility in men. Piecewise linear regression s-segment model shows breaking at the age of 71 years. Rate of decline prior to age 71 is −0.20 degrees per year and −0.80 degrees per year after the age of 71 years. (*R* ^2^ of fit *R* ^2^ = 0.09). (b) Age analysis for shoulder flexibility in women. Piecewise linear regression s-segment model shows breaking at the age of 63 years. Rate of change prior to age 63 is 0.38 degrees per year and −0.74 degrees per year after the age of 63 years. (*R* ^2^ of fit *R* ^2^ = 0.09).

 The mean range of motion of shoulder flexibility was 138 degrees in our sample, with no difference between men and women. Shoulder abduction showed a significant decline across age, averaging 5 degrees per decade in men and 6 degrees per decade in women. From piecewise linear regression, an accelerated decline of 0.80 degrees per year was observed in the sample of men starting with those 71 years old, whereas in women the onset of decline was 63 years and declined across age at a rate of 0.74 degrees per year (Figures 1(a) and 1(b)). 

#### 3.3.2. Hip Flexion

**Figure 2:**
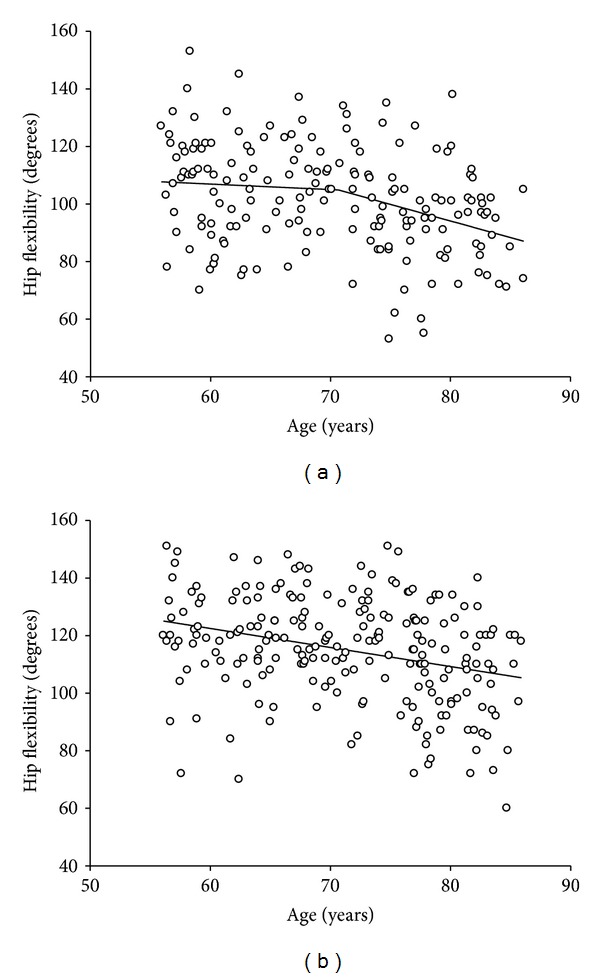
(a) Age analysis for hip flexion in men. Piecewise linear regression s-segment model shows breaking at the age of 71 years. The rate of decline prior to 71 years is −0.19 degrees per year and −1.16 degrees per year thereafter. (*R* ^2^ of fit *R* ^2^ = 0.11). (b) Age analysis for hip flexion in women. Piecewise linear regression s-segment model shows breaking at the age of 86 years. The rate of decline prior to 86 years is −0.66 degrees per year and −2.67 degrees per year thereafter. (*R* ^2^ of fit *R* ^2^ = 0.08).

 The women had significantly higher hip flexion of 114 degrees versus the men, with 102 degrees. However, both showed a similarly significant age-related decline in hip flexion (men: 6 degrees per decade; women: 7 degrees per decade). Piecewise linear regression revealed a rate of decline of 1.16 degrees per year, across age, beginning at 71 years in men (Figure 2(a)). In women, the decrease across the age span of the sample was a single linear decline of 0.66 degrees per year (Figure 2(b)).

### 3.4. Relationship of Age and Physical Activity with Flexibility

Both upper and lower body flexibility measures were normally distributed. Age was significant (*P* < 0.01), but the contribution of physical activity was not (females: *P* = 0.14; males: *P* = 0.57), when included in a regression model that described 9% of the variance for both males and females in the decline in shoulder abduction. The regression model accounted for only 7% of the variance (in both men and women) in the change in hip flexion. Again, age showed a significant contribution (*P* < 0.01); however, the contribution of physical activity to lower body flexibility was not significant for either males (*P* = 0.71) or females (*P* = 0.42). 

### 3.5. Variables Associated with Flexibility

Neither total physical activity nor the components of light-, moderate-, and heavy-intensity physical activity were significantly related to flexibility of the hip or shoulder at the univariate level (Tables 2(a) and 2(b)). Age was significant and explained 8% and 7% of variance in shoulder and hip flexibility, respectively.

For upper body flexibility, age, BMI, plantar flexor strength, and handgrip strength were entered into the stepwise linear regression (Table 3(a)). Regression analysis yielded a model including age, BMI, and plantar flexion strength that resulted in 10.5% of the variance in upper body flexibility being accounted for by those variables.

Age, sex, BMI, and hand grip strength were entered into a regression model for lower body flexibility, accounting for 19.6% of the variance in hip flexibility (Table 3(b)).

### 3.6. Association with Function, Self-Rated Health, and Life Satisfaction

There was no association between upper body flexibility and the “functional measures” of self-reported difficulty in walking or climbing stairs. Step length was associated with upper body flexibility but not when adjustment was made for age. Normal, fast, and very fast walking speeds were associated with upper body flexibility, but only very fast walking speed (*P* = 0.001) was still associated when adjustment was made for age. Lower body flexibility was associated with all walking speeds; however, none of the associations were maintained when adjustment was made for age.

Self-rated health and life satisfaction were not associated with either upper (*P* = 0.18; *P* = 0.32) or lower body flexibility (*P* = 0.09; *P* = 0.30).

## 4. Discussion

This study provides descriptive data on the age-related differences (across the age range of 55–85 years) in flexibility in a large cross-sectional sample of male and female community-dwelling older adults. It also provides an examination of the role of physical activity in the changes to upper and lower body flexibility with aging and a determination of the relationship of flexibility with functional outcomes in older adults. Our sample demonstrated a mean upper body flexibility of 138 degrees and a mean lower body flexibility of 109 degrees. Bassey et al. [12] reported shoulder abduction values of 125 degrees for men and 119 for women in a similar large sample (*n* = 894) of community-dwelling adults over the age of 65 years. These values are lower than those reported for the present study's sample; however, it should be noted that the shoulder abduction measure was slightly different, and a large proportion of the sample in Bassey's study reported having a functional disability.

 With respect to sex differences, the majority of the literature indicates that women have greater flexibility at all ages [4, 13–18]. Our results were in agreement for lower body flexibility, although there was no significant difference based on sex for upper body flexibility. This is in contrast to Bassey et al. [12], who reported significantly lower shoulder abduction flexibility for females in their sample. Doriot and Wang [19] did not find consistent sex differences among their 26 measures of joint range of motion. Similarly, Walker et al. [20] found no differences in ranges of motion of the shoulder, elbow, hip, or knee joints, between older men and women. These varying results are likely due to different population samples, joints studied, and customary use of the joints.

The rate of decline in flexibility with age will vary depending on the body part measured, the training status of the sample, and population being studied. In our sample of relatively healthy community-dwelling older adults, the rate of decline in our measure of upper body flexibility (shoulder abduction) was 0.5 degrees per year in males and 0.6 degrees per year in females. Declines in hip flexion of 0.6 degrees per year in males and 0.7 degrees per year in females were documented. A 1% decline per year (approximately 1.2 degrees per year, or nearly double the rate found in the present study) in shoulder abduction range of motion of older men and women was reported by Bassey et al. [12]. Comparative rates of decline are not readily available in the literature, but rates of 1.5 degrees per year have been reported for lower back flexion, and the greatest decline appears to occur with trunk extension [21].

Whereas differences in flexibility by sex may occur, the rate of change with age has been reported to be similar in both men and women [22, 23], and our results concur. In contrast, McCulloch [14] showed little decline in sit-and-reach scores in women versus men, who showed a dramatic decline in age groups of 65 to 75 years, citing differences in the decline in work activity of men over the older adult age range. 

This study provides a description of potential critical periods of decline in flexibility across the older adult age range. At the age of 71 years, it appears that both upper and lower body flexibility show an accelerated decline in males, whereas in females, only upper body flexibility shows a change in the rate of decline, with lower body showing a steady rate of change. James and Parker [22] reported decreases in active and passive motion in lower limb joints during the period of 70 to 92 years, with the decline becoming more pronounced during the ninth decade. While not significant, Charkravarty and Webley [15] reported a greater decline in range of motion in a group over the age of 75 years versus a group of 65–74 years, adding support to the trend for an accelerated decline in flexibility in the oldest old. The present sample had an age range including up to 86 years, and the piecewise linear regression did suggest that an accelerated decline would occur in the oldest women.

Whereas age may be associated with a decline in flexibility, older adults still maintain the ability to improve flexibility with general exercise training programs [24–27] and with flexibility-specific training, as reviewed by Stathokostas et al. [1]. In addition, the difference in rate of change in flexibility across joints has been attributed to chronic use of those joints, for example, those used in activities of daily living. As such, one purpose of the present study was to determine if age-related losses in flexibility were associated with in physical activity levels. Our results showed no relationship between self-reported physical activity levels and upper or lower body flexibility. Walker et al. [20] also reported no differences in the ranges of motion in the shoulder, elbow, hip, or knee joints, in a sample of 60 older men and women classified into high and low physical activity categories based on self-report. Also, similar results were found by Miotto et al. [28] when comparing the hamstring flexibility in a sample of active versus sedentary adults with a mean age of 68 years; no difference was observed. Bassey et al. [12] studied the association between shoulder abduction and self-reported customary use of the shoulder and found an association; however, it should be noted that the effect was not significant in women in multiple regression (replaced by effort score), and the effect of customary use was greater in those with a disability. This finding may suggest that a more closely-matched flexibility and activity-specific measurement is more reflective of the role of physical activity in the change in flexibility with age. Nevertheless, in a smaller sample of 30 older women, Rikli and Busch [29] found a significant difference for trunk and shoulder flexibility in active versus inactive women, where active was considered as vigorous activity for at least 30 minutes, three days per week. This study reported a significant age-by-activity interaction for shoulder flexibility, but not for trunk flexion. Voorrips et al. [30], in a sample of 50 women with a mean age of 72 years, reported significantly better flexion at the hip and spine in women who self-reported high activity levels (several hours per week in aerobic-type exercises). A five-year longitudinal study by Lan et al. [31] demonstrated that baseline and follow-up thoracolumbar flexibility values were higher in older adults participating in a Chinese conditioning program of repeated motions and postures with range of motion warm-up versus a sedentary control group. Further, while both groups showed an age-related decline over the five years, the control group had a larger decline in flexibility, supporting a positive role of physical activity in attenuating the decline in flexibility with age. Thus, our results suggest that the age-related declines in flexibility of disability-free independently living older adults are not influenced by their overall level of daily physical activity (although specific stretching exercises can still alter the flexibility levels of older adults).

The present study also examined whether shoulder or hip flexibility was related to “functional” outcomes, specifically walking speeds or self-reported mobility difficulty. Normal step length and normal, fast, and very fast walking speeds were associated with shoulder abduction; however, only for very fast walking speed was the association consistently maintained when adjustments were made for age. Our results did not provide evidence that the change in lower body flexibility (hip flexion) impacted functioning with age. Normal, fast, and very fast walking speeds were associated with hip flexion, but as with shoulder abduction, the relationship was not sustained when adjustment for age was made. There was no association with self-reported difficulty in walking. A factor to consider in range-of-motion declines is the loss of compliance in connective tissue with aging. This loss can lead to decreased range of motion and therefore mobility limitations. For example, it was shown by Vandervoort et al. [32] that a loss of flexibility in the ankle joint affects walking mechanics. It might have been expected that our measure of lower body flexibility would be associated with our walking measures, as representatives of function; however this was not the case. This may be due to the lack of contribution of hip flexion to gait. Nevertheless, self-reported difficulty with stair climbing also failed to show an association in the present population. Previously, our laboratory identified shoulder flexibility as one determinant of independence when comparing a group of independently living older adults versus those in rest or nursing homes [33]. Tainaka et al. [34] showed that ankle dorsi-flexion range of motion was a significant physical fitness factor in predicting six-year incidence of disability. These studies might suggest that the roles of flexibility and function with aging are population-dependent and may not be as influential in younger or healthy subgroups of older adults, based on epidemiological data. Nevertheless, based on the reference values indicating that shoulder abduction range of motion of 120 degrees and hip flexion values of 30–50 degrees (for most hip-related functional activities) are considered lower-end thresholds associated with functional loss [35], we would consider our sample of healthy community-dwelling older adults to be high functioning. Based on the present data for shoulder abduction, using the “reference” that a value of <120 degrees was related to functional loss, the conclusion would be that, among our community-dwelling, disability-free sample the probability of the age-related decline in flexibility falling to below the reference values was very low—less than ~10 subjects beyond age 75 years fell below this “functional threshold” and the average for the 85 year old was close to 130 degrees. For the hip flexibility measure of the present study, we are not aware of data to establish a functional threshold; however from the present data where hip flexion was not related to functional outcomes, the hip flexion was above 70 degrees and the average for the 85 year old was ~100 degrees. 

An individual's quality of life includes their sense of well-being, which depends on how they feel about their health and their level of satisfaction with life. In order to address the broader issue of how physical fitness attributes can contribute to health in older adults, the relationship between these health indicators and flexibility was examined. Self-rated health and life satisfaction were not associated with either upper or lower body flexibility in the present sample of independent older adults. In contrast, Bassey et al. [12] reported an association of life satisfaction and social engagement with shoulder range of motion in a large sample of older men and women. However, the difference between studies, as mentioned earlier, is that the sample of Bassey et al. [10] reported a high rate of disability, including shoulder-specific disability and arthritis. In our sample, no relationship between arthritis and flexibility was indicated. In support of the decline in flexibility playing a role in quality of life of older adults, Fabre et al. [36] reported a significant association between upper body flexibility and health-related quality of life in nonagenarians. This sample was community dwelling, with 45% of the sample reporting orthopedic conditions and 43% reporting at least one chronic condition. Thus, although further research is required to understand the role of flexibility in quality of life and successful aging, a lack of relationship is suggested from our data, and where an association of flexibility and health outcomes occurs, it is likely related to a disability, that is, a range of joint motion below some critical threshold.

### 4.1. Limitations

While the present study does describe a large number of men and women from a random sample, the data is cross sectional, and so reverse causality cannot be ruled out. In addition, individual trajectories of flexibility could vary due to the individuality of the aging process, which would be provided by longitudinal data. The joints measured and the functional outcomes may not be tightly matched or may not reflect functions of daily living that could potentially be limited in subgroups of the present sample, or in the older age ranges. Further to this point, based on the inclusion criteria for this study, the sample may not be representative of the “usual” aging population, but rather an independently living generally healthy one. 

## 5. Conclusions

A decrease in flexibility of the shoulder and hip joints by approximately 6 degrees per decade was observed across ages 55 to 86 years in both men and women. Analysis of age subgroups shows that both shoulder and hip joints begin to experience significant declines after 70 years. Physical activity level did not explain a significant amount of the variance in flexibility measures, and flexibility was not associated with functional ability. While steeper gradients of flexibility with age over certain thresholds may be indicated, further analysis is warranted to discern whether the losses in flexibility impact functional outcomes and the degree of loss of range of motion that might relate to disability. In particular, a more direct matching of specific limb range of motion and meaningful functional outcome is needed, as are longitudinal studies. Additionally, the specific type of physical activity that may influence the age-related loss needs to be further elucidated. Nevertheless, overall, in community-dwelling generally healthy older adults aged 55 to 85 years, the age-related loss of flexibility appears to be small such that the normal loss of joint range of motion (i.e., in the absence of underlying clinical condition) is unlikely to neither impact significantly on daily functions nor result in disability. 

## Figures and Tables

**Table 1:** Subject characteristics.

	Total sample (*n* = 436)	Men (*n* = 205)	Women (*n* = 231)
Age (years)	71 ± 8.6	70.4 ± 8.8	71.4 ± 8.4
BMI	26 ± 3.9	26.3 ± 3.1	25.8 ± 4.4
Mass (kg)	71 ± 12.9	78.2 ± 10.5	64.5 ± 11.4
Physical activity level (MET/minutes/day)	406 ± 201	386 ± 182	423 ± 215
Shoulder abduction (degrees, *n* = 431)	138 ± 15	138 ± 15 (*n* = 202)	138 ± 16 (*n* = 231)
Hip flexion (degrees, *n* = 402)	109 ± 19	102 ± 18 (*n* = 183)	114 ± 18* (*n* = 219)

BMI: body mass index, *P < 0.05.

**Table:** (a)

	Mean (SD)	*r*	*P* value
Sex: males = 205; females = 231		−0.017	0.722
Age (years)	71 ± 8.6	−0.290	<0.001
Total physical activity	405.8 ± 201.0	0.029	0.547
Light activity	186.1 ± 82.6	0.057	0.235
Moderate activity	115.1 ± 91.1	0.030	0.530
Heavy activity	104.6 ± 141.3	−0.012	0.810
BMI	26 ± 3.9	−0.094	0.052
Sum of skinfolds	56.0 ± 20.5	0.013	0.825
Plantar flexion strength	879.5 ± 322.9	0.197	<0.001
Handgrip strength	292.1 ± 112.7	0.126	<0.001
Arthritis: no = 107; yes = 108		−0.022	0.752

BMI: body mass index; self-reported arthritis data available for 215 subjects.

**Table:** (b)

	Mean (SD)	*r*	*P* value
Sex: males = 205; females = 231		0.341	<0.001
Age (years)	71 ± 8.6	−0.256	<0.001
Total physical activity	405.8 ± 201.0	0.027	0.586
Light activity	186.1 ± 82.6	0.039	0.435
Moderate activity	115.1 ± 91.1	−0.010	0.839
Heavy activity	104.6 ± 141.3	0.022	0.658
BMI	26 ± 3.9	−0.131	0.009
Sum of skinfolds	56.0 ± 20.5	0.080	0.187
Plantar flexion strength	879.5 ± 322.9	0.055	0.279
Hand grip strength	292.1 ± 112.7	−0.113	0.029
Arthritis: no = 107; yes = 108		−0.107	0.132

BMI: body mass index; self-reported arthritis data available for 215 subjects.

**Table:** (a)

	*R* ^2^	Parameter estimate	SE	*P* value
Age	0.083	−0.486	0.091	<0.001*
BMI	0.009	−0.647	0.187	0.001*
Plantar flexion strength	0.039	0.006	0.002	0.045*

Cumulative *R* ^2^ = 0.117, BMI: body mass index, *significance set at = *P* < 0.05.

**Table:** (b)

	*R* ^2^	Parameter estimate	SE	*P* value
Age	0.066	−0.571	0.117	<0.001*
Sex—female	0.117	18.8	2.721	<0.001*
BMI	0.09	−1.014	0.265	<0.001*
Handgrip strength	0.029	0.031	0.013	0.018*

Cumulative *R* ^2^ = 0.235, BMI: body mass index, *significance set at = *P* < 0.05, sex: male = 0; female = 1.
